# Histological and Histomorphometric Evaluation of New Bone Formation after Maxillary Sinus Augmentation with Two Different Osteoconductive Materials: A Randomized, Parallel, Double-Blind Clinical Trial

**DOI:** 10.3390/ma13235520

**Published:** 2020-12-03

**Authors:** Giuseppe Grasso, Stefano Mummolo, Sara Bernardi, Davide Pietropaoli, Giuseppe D’Ambrosio, Giovanna Iezzi, Adriano Piattelli, Serena Bianchi, Enrico Marchetti

**Affiliations:** 1 Dentistry Service, Jewish Hospital, Via Fulda 14, 00148 Roma, Italy; gg453@nyu.edu (G.G.); gdambrosiog@gmail.com (G.D.); 2 Department of Life, Health and Environmental Sciences, University of L’Aquila, 67100 L’Aquila, Italy; stefano.mummolo@univaq.it (S.M.); sara.bernardi@univaq.it (S.B.); davide.pietropaoli@univaq.it (D.P.); serena.bianchi@univaq.it (S.B.); 3 Centre of Microscopy, University of L’Aquila, 67100 L’Aquila, Italy; 4 Department of Medical, Oral and Biotechnological Sciences, University “G. D’Annunzio” of Chieti-Pescara, 66100 Chieti, Italy; giovanna.iezzi@unich.it (G.I.); apiattelli@unich.it (A.P.)

**Keywords:** sinus augmentation, bone allograft, anorganic bovine bone, lyophilized equine bone

## Abstract

This study aimed to investigate the histological features of deproteinized equine bone mineral (DEBM) and anorganic bovine bone (ABB) after human sinus augmentation with the lateral approach. Twenty-three sinus augmentations were performed in 16 patients (male: 10/female: 6) using DEBM or ABB in a randomized fashion. Healing took place over the next 6 months. Bone core biopsies (N = 23) were obtained for each subject prior to placing the dental implants. The biopsies were processed for both histological descriptions and histomorphometric analysis. Statistical analyses were applied as appropriate, defining statistical significance as *p* < 0.05. Core bone biopsies revealed no differences in terms of newly formed bone between groups, or differences in terms of tissue inflammation. Both DEBM and ABB appear to be suitable biomaterials for bone augmentation in sinus lift surgery in the short term. However, dedicated studies are required to confirm these results and their stability in the long term.

## 1. Introduction

The sinus augmentation procedure using the lateral approach is a useful and reliable procedure that allows dental implant placement in edentulous atrophic posterior maxillae when the volume and quality of the bone are insufficient [1]. The major challenges of sinus floor elevation are the quantity of viable bone formation after graft maturation and the long-term survival rate of the dental implants positioned in that region, which depend on the blood supply to the graft and from the cells originating from the bony walls [2,3].

To date, various sinus lift techniques have been proposed and sinus floor elevation has been achieved using various surgical procedures [4,5]. Previous studies support surgical approaches, such as lateral or crestal approaches, with the timing of dental implant placement as either simultaneous or delayed. In addition, according to Chen et al., the presence of biomaterials does not seem to be essential for obtaining the formation of new bone in the maxillary sinus [6]. This concept was further explored by Silva et al. in a systematic review published in 2016, which noted that the dental implant survival rate was 96.00% for sinus augmentation performed without grafting and 99.60% for that with bone graft material [7]. However, following the regeneration triade principle, which relies on the presence of a scaffold, growth factors, and cellular precursors [8], grafting material plays a key role in the success of the sinus lift procedure [9].

Peleg and collaborators indicated that the lateral approach is recommended in patients with a low vertical height of the residual bone and a large volume of the maxillary sinus [10].

In addition, the implant primary stability is the key factor for choosing single- or two-stage surgery with simultaneous or delayed implant placement [9]. When the implant primary stability cannot be guaranteed, the two-step procedure is recommended [11].

To date, research on identifying an ideal graft material has monopolized the scientific literature and several types of material have been proposed, such as autogenous bone, xenografts, allografts, synthetics, and mixtures of various biomaterials [3,9]. In this regard, autogenous bone, owing to its osteoconductive, osteoinductive, and osteogenetic properties [12], seems to show the best performance in the early phase [13]. Unfortunately, the autogenous bone availability is limited and requires extra donor site surgery, leading to potential extra risks for patients. For these reasons, other biomaterials have been proposed, such as xenografts, allografts, autologous platelet concentrates, and autologous dentine derivates [14,15,16,17].

As stated by Burchard, the ideal graft material should be osteo-inductive, absorbable, easy to handle, and available in large quantities [18]. Autogenous bone is the first choice in terms of both osteoconductive and osteo-inductive properties; however, intra- or extraoral harvesting may expose patients to increased morbidity risks due to the need for double surgery [19,20]. Sinus floor augmentation through a bone substitute is less invasive than autogenous bone and shows comparable results in terms of implant survival rates [11,21]. Today, a multitude of bone substitute materials with demonstrated acceptable outcomes in terms of biocompatibility and long-term stability are available from several specialized companies [22,23], and several investigations on anorganic bovine bone (ABB) [15,24,25,26] and deproteinized equine bone mineral (DEBM) have been conducted [27,28,29,30].

DEBM is a heterologous material harvested from equine specimens and after several chemical-enzymatic steps, the collagen component and the mechanical resistance are preserved. This condition leads to a total remodeling process which allows substitution with endogenous bone. Conversely, ABB is composed of bovine-derived hydroxyapatite that has been deproteinized with a two-step sterilization procedure, ensuring decontamination from bacteria and viruses. It is a non-resorbable material that ensures bone defect filling. Moreover, its macro geometry with micro and macro pores allows vascular ingrowth and stability.

The ABB class of grafting biomaterial exhibits osteoconductive and biocompatibility properties, making it suitable for regeneration procedures, such as socket preservation [31]. However, the obtaining process, which includes stages of deproteinization followed by heat treatment, does not exclude the possibility of disease transmission, such as bovine spongiform encephalopathy [32].

Therefore, other animal sources have been investigated, such as horse species; the bone grafts obtained by horse bone (DEBM) did not show any immune reactions, as previously reported, and displayed osteoconduction and biocompatibility properties [33].

Nevertheless, to the best of the authors’ knowledge, only a few comparative histological or histomorphometric studies on DEBM and ABB in human sinus augmentation have been carried out [34,35].

The aim of this study was to investigate and compare the histological and histomorphometric characteristics of DEBM and AAB used for the maxillary sinus augmentation procedure with the lateral approach.

## 2. Materials and Methods

### 2.1. Study Design

The study was a randomized, double-blind, parallel human clinical trial that aimed to evaluate the clinical and histological features of DEBM (Bio-Gen mix, Bioteck, Arcugnano, VI, Italy) and ABB (Endobon, Zimmer Biomet Dental, Palm Beach Gardens, FL, USA) in sinus lift surgery using the lateral approach. Both materials’ particles have the same size (0.5–1 mm granules). The graft material was unknown for the patients and for the researcher performing histological procedures (G.I.), but not for the surgeon (G.G.).

All procedures were performed in accordance with the Declaration of Helsinki and the Good Clinical Practice Guidelines.

The present study was approved by the Ethics Committee of the Jewish Hospital of Rome (Protocol 4099/7DG/O.I.) and was registered at clinicaltrials.gov (NCT02865590). All study participants gave their informed consent prior to study enrollment.

### 2.2. Patient Selection

Caucasian patients aged ≥25 years with at least one atrophic posterior maxilla with a residual crest height ≤ 4 mm who needed maxillary sinus lift for dental implant placement were eligible for the trial [36,37]. The residual crest height was evaluated by Cone Beam Computed Tomography (CBCT).

Patients with an impaired health/physical status [38], smoking > 10 cigarettes per day, a history of bisphosphonate therapy in the past 3 months, a history of oral and maxillofacial radiant therapy, sinusitis (ETN exam), pregnancy, a recent history of cancer diagnosis (<1 year), obese with a body mass index (BMI) > 30, the presence of auto-immune diseases, or allergies to medications (amoxicillin, clavulanic acid, and ibuprofen) were excluded [38]. Twenty-three (N = 23) consecutive patients were randomly assigned to DEBM or AAB groups by randomization software (STATA/12.1, StataCorp LLC, College Station, TX, USA).

### 2.3. Surgical Procedure

The same operator (G.G.) performed all of the surgical procedures (N = 23) on enrolled patients. Of 23 procedures performed on 16 patients, seven were bilateral. Twelve procedures were performed with DEBM and 11 with AAB. Briefly, crestal and vertical incisions were performed with a 15c surgical blade (Swann-Morton, Sheffield, UK) after local anesthesia (Articaine with adrenaline 1:100.000, Citocartin, Molteni Dental srl, Milan, Italy). The mucoperiosteal flap was raised and a bony window was created on the buccal wall of the maxillary sinus using a round diamond burr mounted on a straight handpiece (40,000 rpm) with sufficient sterile saline cooling. The Schneiderian membrane was gently lifted up, starting from the cranial part, to its complete elevation. Then, the Schneiderian membrane integrity was checked and any perforation was recorded according to Vlassis and Fugazzotto classification [39]. An absorbable collagen membrane (OsseoGuard, Zimmer Biomet Dental, Palm Beach Gardens, FL, USA) was placed to seal perforations when these occurred (N = 3).

Subsequently, the placement of DEBM or ABB occurred according to a randomization group. Graft material was placed in the sinus, avoiding excessive compression. Blood was used to mix materials before their placement. The external window was covered with a double-layer collagen membrane (OsseoGuard, Zimmer Biomet Dental, Palm Beach Gardens, FL, USA) and the mucoperiosteal tension-free flap was repositioned and sutured with 5/0 mono-filament (Monofil, Assut Europe spa, Magliano dei Marsi, AQ, Italy).

### 2.4. Post-Operative Care

Patients received an oral antibiotic every 12 h for 6 days (amoxicillin 875 mg + clavulanic acid 125 mg) and oral anti-inflammatory medications every 8 h for 2 days (ibuprofen 600 mg) after surgery. In addition to standard oral hygiene, a mouth rinse with a 0.12% chlorhexidine solution for 21 days (3 times/day) (Dentosan 0.12%, Recordati SpA, Milan, Italy) was prescribed. Patients were re-examined 10 days after surgery for suture removal. Patients were checked regularly every 30 days in order to verify pain, epistaxis, and rhinorrhea.

### 2.5. Histological Procedure

Six months after the sinus lift procedure, 23 bone biopsies were collected (1 biopsy per sinus augmentation procedure). Ten millimeter (10 mm) height bone biopsies were retrieved from the experimental sites during dental implant placement, using a trephine burr with an internal diameter of 3 mm (Hu-Friedy Manufacturing, Chicago, IL, USA), by the same surgeon who performed the sinus augmentation procedures (G.G.). Biopsy samples were then fixed in 10% buffered formalin. After this phase, the bone cores were processed in order to obtain the ground sections using the Precise 1 Automated System (Assing, Rome, Italy) [40]. Samples were dehydrated with alcohol at different concentrations and embedded in a glycolethacrylate resin (Technovit 7200, VLC, Kulzer, Wehrheim, Germany). After polymerization in the resin, all samples were sectioned with a high-precision diamond disc and then abraded, in order to obtain slides that were about 30 microns thick. Three slices were obtained from each specimen, and were subsequently stained with acid fuchsin and toluidine blue before the analysis. The percentages of new bone, marrow spaces, and residual grafted particles were calculated using a light microscope (Leitz Laborlux, Wetzlar, Germany). The microscope was connected to a high-resolution video camera (3CCD, JVC KY-F55B, JVC, Yokohama, Japan) and a monitor and PC (Intel Pentium III 1200 MMX, Intel, Santa Clara, CA, USA). This optical system was associated with a digitizing pad (Matrix Vision GmbH, Oppenweiler, Germany) and a histometric software package through which the histological images were captured and analyzed (Image-Pro Plus, Media Cybernetics Inc., Immagini e Computer Snc, Milano, Italy) [40]. Histological descriptions and histomorphometric analysis were conducted by a single examiner (G.I.), who was not involved in the surgical treatment.

### 2.6. Statistical Analysis

The Mann–Whitney test for independent variables was used for all of the analyses (STATA/12.1, StataCorp LLC, College Station, TX, USA). Data are presented as the mean ± standard deviation (SD). Statistical significance was set to *p* < 0.05.

## 3. Results

After applying the inclusion/exclusion criteria, 16 consecutive patients (6 females and 10 males), with a mean age of 54 (±7), were enrolled in the study. Twenty-three sinus lift procedures were performed successfully (success rate of 100%). Success criteria were primary stability of the implants through insertion torque > 20 N and a radiographic evaluation of the graft using CBCT with at least 10 mm of radiographic bone in length. Each augmented sinus provided at least one intact core 2 mm in width and 10 to 15 mm in length suitable for the collection of samples. Histomorphometry showed non-statistically significant differences between DEBM and AAB in terms of newly formed bone (Table 1).

### 3.1. DEBM

**Figure 1:**
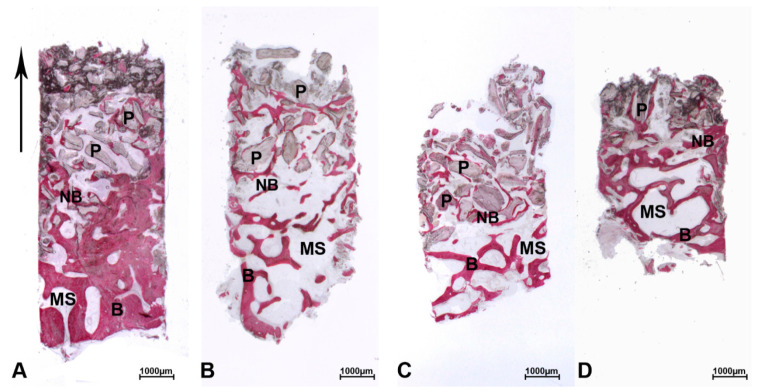
Deproteinized equine bone mineral (DEBM). The biopsies were oriented in the crestal-apical direction (↑) and all biopsies showed a small portion of the pre-existing bone (B) with large marrow spaces (MS). (**A**) In the crestal portion, pre-existing bone (B) with small marrow spaces (MS) and new bone (NB) were present, whilst in the middle portion, the biomaterial particles (P) were gathered but distant and tended to merge in the apical portion. New bone formation (NB) was more present around the particles (P) near the pre-existing bone (B) and less present far from them. (**B**) In the crestal portion, pre-existing bone (B) with large marrow spaces (MS) and new bone (NB) were observed. In the middle portion, the biopsy showed the presence of trabecular bone with large marrow spaces and residual biomaterial (P) which exhibited different sizes in the apical portion. New bone formation (NB) was more present around the particles near the pre-existing bone (B) and less present far from them. (**C**) This biopsy was partially damaged during their removal from the trephine. In the crestal portion, trabecular bone (B) with large marrow spaces (MS) and new bone (NB) were detected. The residual grafted particles (P) were partially encircled by newly formed bone (NB). (**D**) In the crestal portion, the biopsy showed a small portion of pre-existing bone (B) with large marrow spaces (MS) and new bone (NB). Thin trabeculae, wide marrow spaces, and residual DEBM particles scattered in the weave of newly formed bone were present in the apical portion of the sample. Most of the biomaterial granules (P) were partially encircled by new bone (NB). (Toluidine blue and acid fuchsin, original magnification (6×)).

At low magnification, most biopsies of the DEBM group were composed of trabecular bone with large marrow spaces and residual biomaterial particles (Figure 1A–D). Specifically, in the crestal portion of the biopsies, pre-existing and/or newly formed bone was observed. In the apical and middle areas of the samples, residual biomaterial particles could be observed. Indeed, in the middle portion, small trabeculae of newly formed bone were present, forming bridges of bone between the particles. The network of new bone trabeculae connected the residual biomaterial granules.

**Figure 2:**
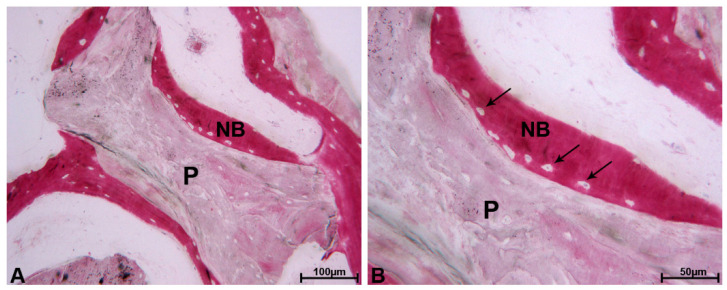
DEBM. (**A**) The residual DEBM particles (P) were encircled by newly formed bone (NB), which was characterized by extensive osteocyte lacunae, and no gaps were evident at the bone biomaterial interface. Toluidine blue and acid fuchsin, original magnification 100×. (**B**) At higher magnification, many osteocyte lacunae (black arrows) at the biomaterial–particle interface (P) were present. Toluidine blue and acid fuchsin, original magnification 200×.

The particles located next to the pre-existing bone of the crestal region were totally or partially encircled by newly formed bone which showed wide osteocyte lacunae, typical of recently mineralized bone (Figure 2A,B).

**Figure 3:**
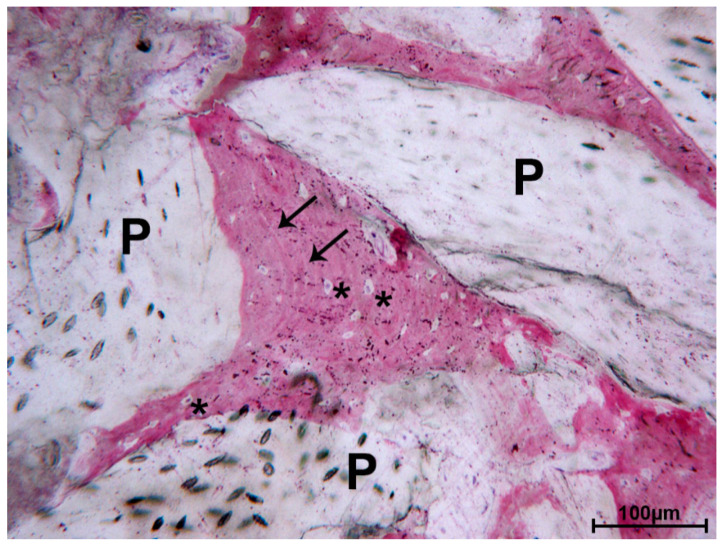
DEBM. Regenerated lamellar bone (black arrows), perfectly filling the inter-DEBM particle (P) gaps. Many osteocyte lacunae were present (*). Toluidine blue and acid fuchsin, original magnification 100×.

In several areas, the bone was in strict contact with the biomaterial and the osteocyte lacunae were in close contact with the particles (Figure 3).

**Figure 4:**
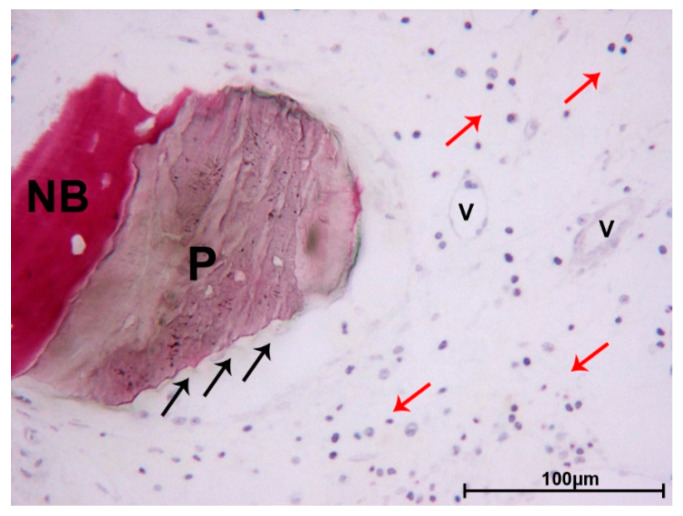
DEBM. Moderate inflammatory infiltrate (red arrows) and small-sized vessels (V) were observed close to a DEBM granule (P), which presented indented margins (black arrows), probably due to a resorption process. A portion of the particle was in contact with newly formed bone (NB). Toluidine blue and acid fuchsin, original magnification 200×.

In the apical portion, the particles of DEBM were only partially surrounded by newly formed bone. In this portion, less bone trabeculae were present between the particles. No multinucleated giant cells or phlogistic cells were present around the residual graft or at the interface with the bone. In one biopsy, a mild inflammatory infiltrate was evident, and some small- and large-sized vessels were observed. The DEBM particle exhibited indented margins, probably due to a resorption process (Figure 4).

Histomorphometry showed that newly formed bone accounted for 22.20%, marrow spaces 50.77%, and the residual DEBM graft 27% of the total volume of the sample (Table 1).

### 3.2. ABB

At low magnification, most biopsies of the ABB group were composed of trabecular bone with marrow spaces and residual biomaterial particles (Figure 1A–C). Indeed, in the crestal portion, pre-existing and/or newly formed bone with large marrow spaces was observed.

**Figure 5:**
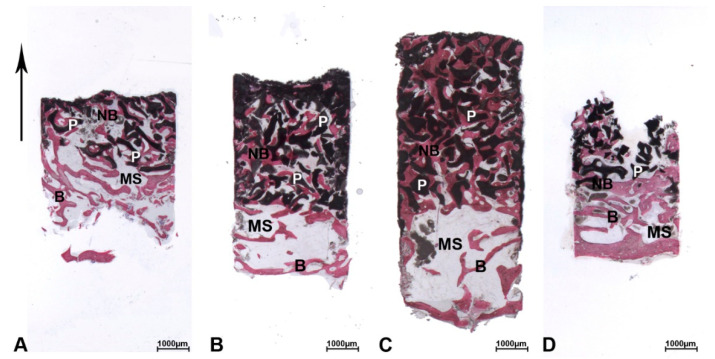
Anorganic bovine bone (ABB). The biopsies were oriented in the crestal-apical direction (↑) and all biopsies showed a small portion of the pre-existing bone (B) with large marrow spaces (MS). (**A**) In the crestal portion, pre-existing bone (B) with large marrow spaces (MS) and new bone (NB) were present. In the middle portion, residual biomaterial particles (P) that appeared more coalescent in the apical portion of the sample were partially encircled by newly formed bone (NB). (**B**) In the crestal portion, pre-existing bone (B) with large marrow spaces (MS) was present. In the middle and apical portions, a huge amount of AAB (P) was still evident in the sample. The particles often converged towards each other and newly formed bone (NB). (**C**) In the crestal portion, pre-existing bone (B) with large marrow spaces (MS) was present. In the middle and apical portions, many residual AAB particles (P) were totally lined by newly formed bone (NB) and small marrow spaces (MS). (**D**) This biopsy was partially damaged during their removal from the trephine burr. In the crestal portion, trabecular bone (B) with large marrow spaces (MS) and new bone (NB) were detected. In the middle and apical portions, residual biomaterial particles (P) were encircled by newly formed bone (NB) and this was more present in the portion close to the pre-existing bone (B). Toluidine blue and acid fuchsin, original magnification 6×.

In the middle and apical portions of the samples, trabecular bone interconnecting and bridging a huge quantity of residual particles of biomaterial was observed (Figure 5A–D). In the middle portion, the residual graft particles were entirely encircled by newly formed bone in the area close to the preexisting bone, thus thickening the crestal bone layer.

**Figure 6:**
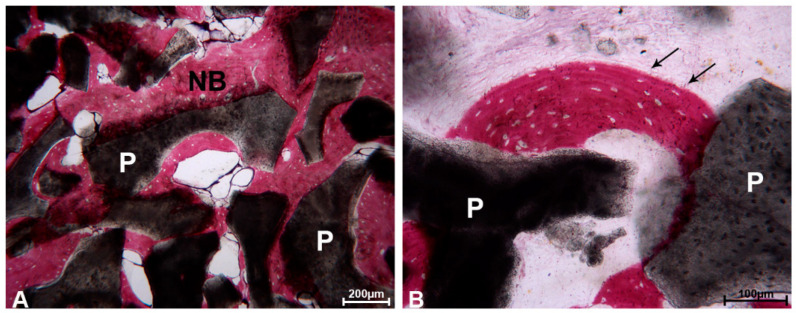
AAB. (**A**) Residual biomaterial particles (P) were encircled by a bridge-like network of newly formed bone (NB). Toluidine blue and acid fuchsin, original magnification 40×. (**B**) At higher magnification, a bridge-like network of newly formed bone (black arrows) between the particles (P) was present. Toluidine blue and acid fuchsin, original magnification 100×.

At high power magnification, the residual graft was encircled by new bone and no spaces were evident at the bone–graft interface. Several particles displayed a lower density at the interface with the new bone, probably due to the beginning of the resorption process (Figure 6A,B).

**Figure 7:**
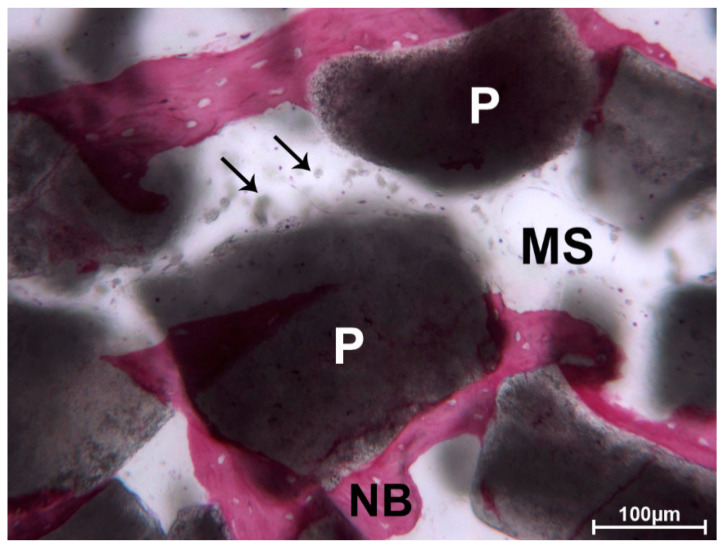
AAB. Large particles of AAB (P) with blurred margins, partially lined by newly formed bone (NB), and small spots of biomaterial (black arrows) colonized the marrow spaces (MS). Toluidine blue and acid fuchsin, original magnification 100×.

In most of the specimens, the graft seemed to undergo resorption; indeed, the marrow spaces were colonized by small spots of biomaterial, with blurred margins (Figure 7).

**Figure 8:**
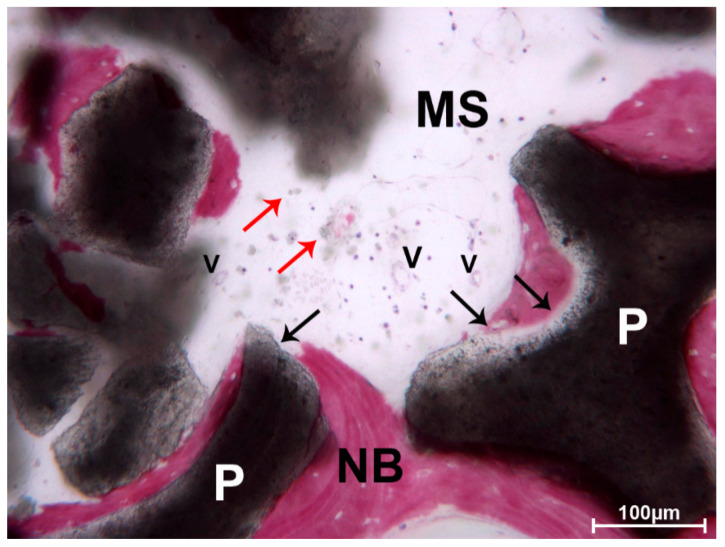
AAB. Mild inflammatory infiltrate and a few small-sized vessels (V) were close to the newly formed bone (NB) surrounding the AAB granules (P). Little spots of biomaterial (red arrows) were scattered in the marrow spaces (MS). The exterior margins of the granules were less dense than their inner portion (black arrows). Residual biomaterial particles (P) was in contact with newly formed bone (NB). Toluidine blue and acid fuchsin, original magnification 100×.

Most of the biopsies did not show phlogistic cells or multinucleated giant cells around the graft material or at the interface with the bone. In two biopsies, a mild inflammatory infiltrate was evident, and a few small- and large-sized vessels were observed (Figure 8).

Histomorphometry showed that newly formed bone represented 22.84%, marrow spaces 46.20%, and the residual graft material 39.94% of the total volume of the sample (Table 1).

## 4. Discussion

This study aimed to evaluate the histological features of DEBM and AAB in human sinus lift procedures performed using the lateral surgical approach. DEBM and AAB are xenografts since they are derived from animals. Even though there are only a few randomized controlled clinical trials comparing DEBM and AAB in the literature [35,36], our results are consistent with previous findings. In fact, both materials share similar characteristics in terms of inflammation and new bone formation, as shown in Table 1. In addition, both materials exhibited graft particles that were in contact with newly formed bone (Figure 3 and Figure 6). Our findings showed that bone regeneration in the DEBM sites was comparable to that in ABB sites (22.20 ± 7.18 vs. 22.84 ± 7.34; *p* > 0.05). Nevins et al. [41] clinically and histologically investigated the use of ABB in 14 maxillary sinus augmentation procedures (Endobon, Zimmer Biomet Dental, Palm Beach Gardens, FL, USA). The mean percentage of newly formed bone at 6 months was 27.5% ± 8.9%, with a slow rate of resorption of the graft. A few years later, Nevins et al. [27] investigated the use of DEBM in maxillary sinus augmentation in a case series study. The histomorphometric analyses revealed a mean formation of the new bone of 23.5% [27]. Rivara et al. [30] reported, in their preliminary histomorphological study, promising results at six-months follow-up, with the significant formation of new bone tissue (39.84% ± 2.96). Stievano et al. [42] performed a retrospective survival study of dental implants positioned in regenerated bone after maxillary sinus augmentation with the Biogen mix, without histological or histomorphometric analysis. The Di Stefano et al. randomized clinical trial (RCT) comparing the two xenografts reported a significantly higher amount of newly formed bone at sites treated with the equine xenograft compared with those treated with the bovine xenograft (46.86% ± 12.81% vs. 25.12% ± 7.25%; *p* < 0.05) at implant placement [34]. Although new bone formation was similar, differences were apparent between ABB sites and DEBM, probably due to the collagenic component of the equine xenograft. Another histological and histomorphometric analysis of six different biomaterials, including ABB and DEBM [35], showed that the equine xenograft had more newly formed bone (22.8%) and lower residual graft material (30.1%) than ABB (16.1% and 37.2%; no statistical analysis was performed). This greater resorption of DEBM could be influenced by the process of deantigenation to which the equine xenograft is subjected [35]. Both studies reported that EDEB was very useful in the sinus augmentation procedure due to new bone regeneration. In our study, however, differences between ABB and DEBM were not statistically significant. As reported by Nevins et al., the maxillary sinus involves “non-natural bone forming” [41]; therefore, the success of the sinus lift intervention is not only due to the right choice of grafting materials, but also to the surgical plan. Indeed, a graft is well-integrated when a good vascular supply can provide the growth factors required for new bone formation [9,27]. In the case of the maxillary sinus, care must be taken in terms of the course of the antral artery to avoid interrupting the arterial blood flow, which is very important for the intervention’s success [43]. Indeed, the antral artery, which represents anastomosis between the superior posterior alveolar artery and the infraorbital artery, courses along the lateral wall of the sinus and might be encountered during the surgical intervention [44]. Even though its interruption might not be life-threatening, integration of the grafting material could suffer from this mistake [43,45]. Within the limitations of the present study in terms of the sample sizes, the qualitative histological and quantitative histomorphometric results of this study demonstrated that the difference between anorganic bovine bone and inorganic equine bone is not statistically significant when they are used alone for maxillary sinus augmentation. Additionally, as shown from the literature review performed, further RCTs with a larger sample size and longer follow-up period should be performed to validate these outcomes.

## 5. Conclusions

The success of maxillary sinus augmentation is represented by the formation of new vital and vascularized bone, suitable for host dental implants. The choice of the right grafting material and a correct surgical plan allow predictable success.

## Figures and Tables

**Table 1:** Histomorphometric analysis: Mean and standard deviations of newly formed bone, residual biomaterial, and marrow spaces of the DEBM and AAB groups are shown. All values are expressed as percentages.

	DEBM (Biogen) (Mean ± SD)	AAB (Endobon) (Mean ± SD)	*p* Value
New bone	22.20 ± 7.18	22.84 ± 7.34	0.6058
Residual biomaterial	27 ± 6.75	30.94 ± 10.20	0.4807
Marrow spaces	50.77 ± 7.59	46.20 ± 14.30	0.2359
